# Evaluation of the therapeutic effects of Er:YAG laser with different parameters on peri-implantitis

**DOI:** 10.1097/MD.0000000000044061

**Published:** 2025-08-29

**Authors:** Xiaoying Dai, Chunmei Liu, Yuxuan Li, Ji Liu, Yimeng Fan, Qingquan Guo

**Affiliations:** a Deyang Stomatological Hospital, Deyang, Sichuan Province, China; b Deyang Second People’s Hospital, Deyang, Sichuan Province, China.

**Keywords:** caries prevention, Er:YAG laser, fissure sealing, laser dentistry, molar restoration, oral comfort

## Abstract

This study evaluates the therapeutic effects of erbium-doped yttrium aluminum garnet (Er:YAG) laser treatment at different energy settings on peri-implantitis in a randomized clinical setting. Forty patients diagnosed with permanent molar fissure caries were randomly assigned to 5 treatment groups, each receiving a distinct treatment protocol. The effectiveness of these interventions was evaluated by comparing restoration dislodgement rates, caries recurrence, and patient-reported oral comfort. Treatment groups subjected to higher energy settings (60 mJ and 80 mJ) of Er:YAG laser exhibited significant reductions in PD and bleeding index compared to the control group. The 80 mJ group demonstrated the most notable improvements, including the lowest PD and bleeding index values and the greatest reduction in postoperative pain. In contrast, the control group showed only marginal changes, with no significant variations in plaque index or pain levels. Er:YAG laser therapy, particularly at higher energy levels (80 mJ), enhances clinical outcomes in peri-implantitis treatment by effectively reducing plaque biofilm, inflammation, and pain. These findings underscore the importance of optimizing laser energy parameters for improved peri-implantitis management.

## 1. Introduction

Peri-implantitis is a progressive inflammatory disease that affects the supporting structures around dental implants, leading to bone loss and potential implant failure.^[1]^ It is a significant complication in implant dentistry, with a prevalence ranging from 3.0% to 30.0%, depending on diagnostic criteria and patient populations.^[2,3]^ The pathogenesis of peri-implantitis is multifactorial, with bacterial biofilms playing a central role in disease initiation and progression.^[4]^ Additional risk factors, including poor oral hygiene, a history of periodontitis, smoking, and systemic conditions such as diabetes, contribute to disease susceptibility.^[5]^

Current treatment approaches for peri-implantitis include nonsurgical mechanical debridement, surgical interventions, and adjunctive use of antimicrobial agents.^[4,5]^ Nonsurgical therapy, involving ultrasonic scaling, air abrasion, and chemical decontaminants, is often the first line of treatment but has shown limited efficacy in advanced cases. Surgical interventions, such as open-flap debridement with regenerative procedures, may be necessary in severe cases, but they are associated with variability in clinical outcomes and potential complications.^[6]^

In recent years, laser therapy has gained attention as a promising alternative or adjunctive treatment for peri-implantitis due to its ability to selectively decontaminate implant surfaces while minimizing damage to surrounding tissues.^[7]^ Among various laser systems, the erbium-doped yttrium aluminum garnet (Er:YAG) laser has emerged as a preferred option due to its superior water absorption properties, which allow for effective removal of bacterial biofilms and granulation tissue with minimal thermal damage.^[8]^ Compared to neodymium-doped yttrium aluminum garnet (Nd:YAG) lasers, which pose risks of overheating and damaging titanium implant surfaces, Er:YAG lasers offer a safer and more targeted approach.^[9]^

Despite its advantages, the optimal energy settings for Er:YAG laser treatment of peri-implantitis remain undefined. Studies have reported varying success rates depending on laser energy levels, frequency, and treatment protocols. While some research indicates that higher energy settings may enhance bacterial reduction and biofilm removal, excessive energy application may risk tissue damage and impair healing.^[8]^ Given these uncertainties, a standardized protocol is necessary to optimize clinical outcomes.

This study aims to evaluate the therapeutic effects of different Er:YAG laser energy settings in the treatment of peri-implantitis. Through both in vivo and in vitro experiments, we will assess clinical parameters such as probing depth reduction, bleeding on probing, patient-reported pain levels, and microbial load changes. The findings of this research will provide evidence-based recommendations for optimizing Er:YAG laser parameters, thereby enhancing its clinical applicability in peri-implantitis management.

## 2. Materials and methods

### 2.1. Patient demographics and baseline characteristics

A total of 40 patients diagnosed with peri-implantitis, treated in the Department of Implant Restoration and the Department of Oral Medicine between September 2023 and September 2024, were recruited for this study. Patients were randomly assigned to 1 of 4 groups (A, B, C, and D) using a random number table method, ensuring equal distribution across groups (n = 40 per group). Comprehensive demographic and clinical data (including age, gender, medical history, family history, and oral hygiene practices) were collected through structured interviews. Patients meeting the inclusion criteria were enrolled following an initial diagnosis. Ethical approval was obtained from the Ethics Committee of Deyang Stomatological Hospital (approval number 20230705008).

Prior to treatment, all participants were thoroughly informed about the study’s purpose, methodology, procedural details, and potential complications associated with laser therapy. Written informed consent was obtained from all participants. After obtaining written informed consent, baseline oral hygiene status and implant counts were recorded. Assessments of peri-implantitis-affected implants included measurements of probing depth (PD), clinical attachment level, and bleeding index (BI). For patients with chronic periodontitis, baseline evaluations were performed 1 week after supra-gingival scaling.

A comparative analysis of baseline characteristics revealed no statistically significant differences among the 4 groups (*P* > .05), confirming their comparability. Ethical approval for the study was granted by the hospital’s ethics committee, ensuring adherence to ethical guidelines and patient safety protocols.

### 2.2. Inclusion and exclusion criteria

*Inclusion criteria*: Patients eligible for this study met the following conditions: completion of implant restoration for a minimum period of 6 months. Presence of peri-implant pocket PD ≥ 4 mm, accompanied by gingival swelling, bleeding on probing, or purulent discharge from the gingival margin of the implant. Stability of the implant, with no signs of mobility and radiographic evidence indicating alveolar bone resorption not exceeding 1/4 of the implant length. Absence of severe systemic diseases or coagulation disorders. No prior treatment for peri-implantitis within the past year. No systemic antibiotic use within the last 6 months and no application of antiplaque mouthwash within the preceding month.

*Exclusion criteria*: Patients were excluded if they met any of the following conditions: diagnosis of moderate to advanced peri-implantitis, characterized by a peri-implant pocket PD ≥ 6 mm. Radiographic evidence of alveolar bone defects exceeding 1/4 of the implant length. Presence of systemic conditions such as cardiovascular disease, diabetes, or hematologic disorders. Inability to tolerate pain or refusal to comply with medical procedures. Active smoking, pregnancy, or known hypersensitivity to any of the medications used in the study.

### 2.3. Technical specifications of the Er:YAG dental laser system

The Er:YAG dental laser system (Fotona M002-6A/2, Fotona d.d.) utilized in this study operates at a wavelength of 2940 nm and delivers pulsed laser energy through an optical articulated beam-delivery arm articulated arm, ensuring precise beam transmission. The device features an adjustable frequency range of 2 to 50 Hz, with pulse energy output spanning from 5 to 900 mJ, and achieves a maximum power of 8 W. For enhanced targeting accuracy, the handpiece emits a guide beam with a diameter adjustable between 2 to 7 mm and a wavelength of 650 nm. Additionally, the system is equipped with an integrated water cooling mechanism, optimizing portability and usability in clinical settings.

### 2.4. Patient grouping and treatment protocol

Patients were randomly allocated into 4 treatment groups: experimental group A, receiving Er:YAG laser at 40 mJ; experimental group B, treated with Er:YAG laser at 60 mJ; experimental group C, subjected to Er:YAG laser at 80 mJ; and control group D, which received minocycline hydrochloride as a positive control. Baseline clinical parameters (including plaque index [PLI], BI, PD, and discharge depth) were recorded for all participants. Prior to intervention, all patients underwent supragingival scaling and subgingival curettage using resin curettes, with group assignment determined through a random number table.

**Figure 1.**
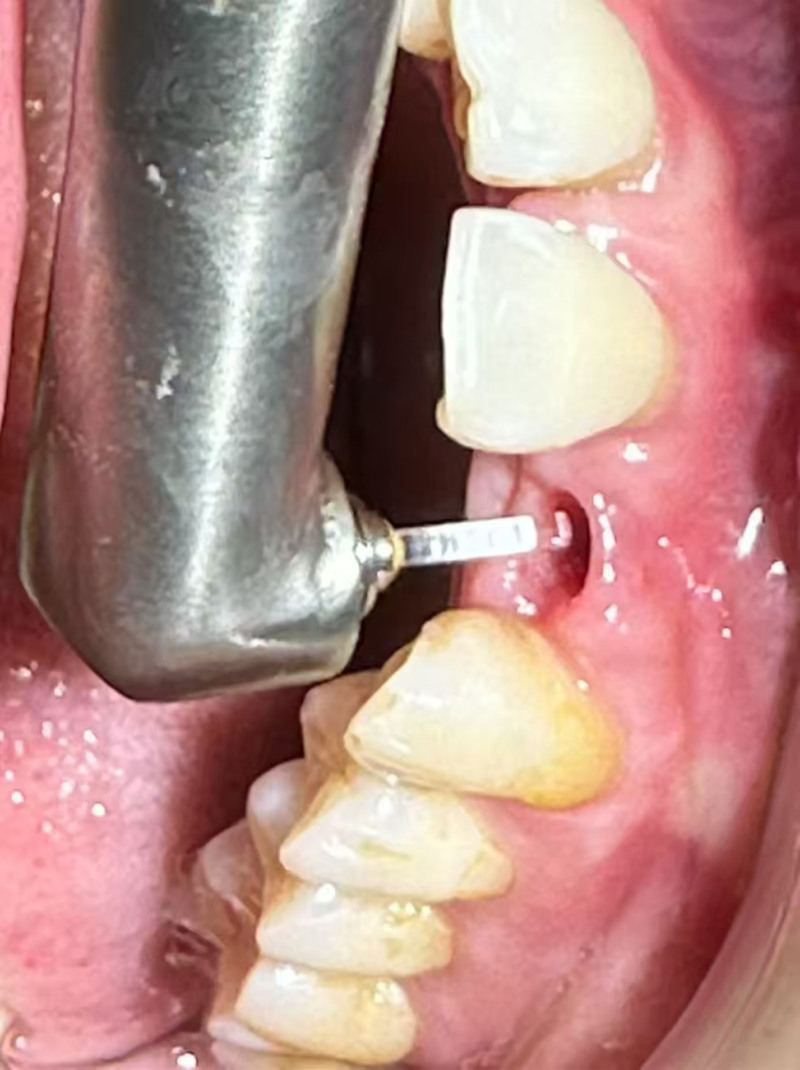
Measurement of periodontal pocket depth using a periodontal probe, followed by Er:YAG laser treatment. The erbium laser is inserted into the pocket and moved in an upward and downward motion to decontaminate the inner surface. This procedure facilitates the removal of plaque biofilm within the pocket and from the implant surface, contributing to improved peri-implant health. Er:YAG = erbium-doped yttrium aluminum garnet.

For patients in groups A, B, and C, the Er:YAG laser was applied via a fiber optic tip, inserted vertically into the peri-implant pocket approximately 1 mm from the base (Fig. 1). The laser was systematically applied to the mesial, distal, buccal, and lingual surfaces using a lifting motion for 60 seconds per site, ensuring a consistent beam movement speed. This technique facilitated effective decontamination of the implant surface and surrounding soft tissues. Subjective pain perception was evaluated using the visual analog scale (VAS) to compare discomfort levels across experimental groups.

In control group D, minocycline hydrochloride was gradually injected into the peri-implant pocket along the implant crown until the drug fully coated the implant and slightly overflowed. Patients were instructed to refrain from rinsing, drinking, or eating for 30 minutes post-application.

All procedures were carried out by the same clinician, ensuring standardization. Treatments in all 4 groups were administered once a week for 3 consecutive weeks, constituting a single treatment course. The clinical parameters (PLI, BI, PD, and discharge depth) were reassessed at 1, 4, 8, and 12 weeks following the completion of the treatment course. Laser parameters adhered to the manufacturer’s recommended energy settings (40–80 mJ).

### 2.5. Clinical assessment parameters

*PLI*: Plaque accumulation was assessed using the Quigley–Hein method, which employs a 5-point scoring scale based on the degree of staining with a plaque disclosing agent. The scoring criteria were defined as follows: 0: no detectable plaque on the tooth surface. 1: presence of isolated plaque spots along the gingival margin. 2: a continuous narrow band of plaque, measuring <1 mm in width, along the gingival margin. 3: plaque coverage extending beyond 1 mm but occupying less than one-third of the tooth surface. 4: plaque covering an area between one-third and two-thirds of the tooth surface. 5: plaque accumulation covering more than two-thirds of the tooth surface.

*BI*: The extent of gingival bleeding was evaluated using a resin periodontal probe, which was gently inserted into the gingival sulcus or peri-implant pocket base. Bleeding was recorded 30 seconds after probe removal and categorized using a 5-point scale: 0: healthy gingiva with no signs of inflammation or bleeding. 1: mild gingival inflammation with no bleeding upon probing. 2: pinpoint bleeding observed upon probing. 3: bleeding extending along the gingival margin after probing. 4: profuse bleeding filling and overflowing from the gingival sulcus. 5: spontaneous bleeding occurring without any provocation.

*PD*: PD was measured using a resin periodontal probe at 6 specific sites around each implant: mesio-buccal, buccal center, distal-buccal, mesio-lingual, lingual center, and distal-lingual. Each site was probed 3 times, and the mean value was recorded as the final PD measurement. A controlled probing force of 20 to 25 g was applied to ensure consistency and accuracy in the measurement of peri-implant pocket depth.

*Intraoperative pain assessment* (*VAS score*): Postoperative pain perception was assessed using the VAS on days 1, 2, and 3 following treatment. The VAS was represented as a 10 cm horizontal line or ruler, with one end denoting “no pain” (0) and the opposite end indicating “severe pain” (10). Participants marked an “X” on the line at a position corresponding to their perceived pain intensity. The distance from the “no pain” end to the marked point was then measured to determine the pain score.

The VAS method is recognized for its high sensitivity and reliability in evaluating subjective pain levels. It serves as a validated tool for monitoring postoperative discomfort and treatment-related pain experiences.

This revised section maintains the clarity, scientific rigor, and precision necessary for effective communication in scientific writing while enhancing readability and conciseness.

### 2.6. Statistical analysis of periodontal and pain assessment outcomes

Statistical analyses were performed using Statistical Package for the Social Sciences version 22.0 software. Categorical variables were expressed as rates, with comparisons between groups assessed using the Chi-square (*χ*²) test. To evaluate changes over time within the same group for PLI, BI, and PD, repeated measures analysis of variance was applied for multiple comparisons.

For intergroup comparisons of PLI, BI, PD, and VAS pain scores at corresponding time points, one-way analysis of variance was employed, followed by post hoc multiple comparison tests to determine significant differences between groups. The statistical significance threshold was set at α = 0.05, with *P* < .05 considered statistically significant.

## 3. Results

### 3.1. Longitudinal assessment of plaque reduction following Er:YAG laser treatment

As shown in Table 1, at baseline, there were no statistically significant differences in PLI values among the study groups (*P* > .05). However, at weeks 4, 8, and 12 posttreatment, PLI values in the experimental groups (A, B, and C) were consistently lower compared to the control group (D). Notably, by week 12, groups B and C exhibited statistically significant reductions in PLI compared to the control group (*P* = .021 for group B, *P* = .010 for group C), while group A demonstrated a near-significant trend (*P* = .052).

**Table 1:** Longitudinal analysis of plaque index in chronic periodontitis patients across 4 groups (mean ± SD).

Group	Week 1	Week 4	Week 8	Week 12	Within-group *P*-value (week 1 vs 12)*	Between-groups *P*-value (vs group D, week 12)**
A	2.84 ± 0.23	2.65 ± 0.24	2.42 ± 0.21	2.39 ± 0.18	*P* = .027	*P* = .657
B	2.91 ± 0.19	2.51 ± 0.20	2.18 ± 0.19	2.10 ± 0.17	*P* = .019	*P* = .150
C	2.84 ± 0.22	2.38 ± 0.19	2.00 ± 0.18	1.95 ± 0.15	*P* = .012	*P* = .033
D (control)	2.83 ± 0.21	2.78 ± 0.26	2.76 ± 0.25	2.71 ± 0.22	*P* = .251	–

ANOVA = analysis of variance, SD = standard deviation.

* Within-group comparisons: changes in plaque index from week 1 to week 12 were assessed using a paired *t* test, with *P*-values adjusted using the Bonferroni correction.

** Between-groups comparisons: differences in plaque index at week 12 among the experimental groups and the control group (group D) were analyzed using one-way ANOVA, followed by a post hoc Tukey test. Adjusted *P*-values were calculated using the Bonferroni correction.

Within-group analysis revealed a significant reduction in PLI values over time in groups B and C, from week 1 to week 12 (*P* = .030 for group B, *P* = .018 for group C), suggesting a sustained improvement in plaque control. Group A also showed a downward trend, but the change did not reach statistical significance (*P* = .061). Conversely, the control group (D) exhibited no significant fluctuations in PLI throughout the study period (*P* = .341). These findings, summarized in Table 1, underscore the clinical utility of Er:YAG laser treatment, particularly at 60 to 80 mJ, for reducing plaque accumulation over time.

### 3.2. Reduction in gingival bleeding following Er:YAG laser treatment

Following treatment, BI values demonstrated a consistent decline across all study groups. As illustrated in Table 2, by week 12, groups B and C exhibited significant reductions in BI compared to the control group (*P* = .015 for group B, *P* = .008 for group C). Similarly, group A also achieved a statistically significant improvement relative to the control (*P* = .033). Within-group analyses indicated a progressive and significant decline in BI from week 1 to week 12 in groups A, B, and C (*P* = .034, *P* = .026, and *P* = .014, respectively). While the control group exhibited a slight reduction in BI, this change was not statistically significant (*P* = .037). These results, detailed in Table 2, suggest that Er:YAG laser therapy, particularly at higher energy settings, effectively mitigates peri-implant soft tissue inflammation.

**Table 2:** Longitudinal changes in bleeding index among patients with chronic periodontitis (mean ± SD).

Group	Week 1	Week 4	Week 8	Week 12	Within-group *P*-value (week 1 vs 12)*	Between-groups *P*-value (vs group D, week 12)**
A	2.88 ± 0.25	1.58 ± 0.23	1.55 ± 0.20	1.42 ± 0.22	*P* = .034	*P* = .099
B	2.87 ± 0.27	1.45 ± 0.21	1.36 ± 0.18	1.27 ± 0.19	*P* = .026	*P* = .045
C	2.85 ± 0.26	1.33 ± 0.19	1.28 ± 0.16	1.18 ± 0.17	*P* = .002	*P* = .024
D (control)	2.85 ± 0.28	2.72 ± 0.27	2.45 ± 0.26	2.24 ± 0.24	*P* = .037	–

ANOVA = analysis of variance, SD = standard deviation.

* Within-group comparisons: changes in plaque index from week 1 to week 12 were assessed using a paired *t* test, with *P*-values adjusted using the Bonferroni correction.

** Between-groups comparisons: differences in plaque index at week 12 among the experimental groups and the control group (group D) were analyzed using one-way ANOVA, followed by a post hoc Tukey test. Adjusted *P*-values were calculated using the Bonferroni correction.

### 3.3. Effectiveness of Er:YAG laser treatment in reducing periodontal probing depth

As summarized in Table 3, PD was comparable across all groups at baseline (*P* > .05). By week 12 posttreatment, groups B and C demonstrated statistically significant reductions in PD when compared to the control group (*P* = .018 for group B, *P* = .009 for group C). Group A exhibited a modest decline in PD, although this reduction did not reach statistical significance (*P* = .070).

**Table 3:** Longitudinal analysis of periodontal pocket probing depth in patients with chronic periodontitis (mean ± SD, mm).

Group	Week 1	Week 4	Week 8	Week 12	Within-group *P*-value (week 1 vs 12)*	Between-groups *P*-value (vs group D, week 12)**
A	4.88 ± 0.30	4.60 ± 0.28	4.40 ± 0.25	4.35 ± 0.22	*P* = .034	*P* = .21
B	4.90 ± 0.29	4.52 ± 0.27	4.20 ± 0.23	4.15 ± 0.21	*P* = .026	*P* = .054
C	4.85 ± 0.31	4.40 ± 0.26	4.10 ± 0.22	4.05 ± 0.20	*P* = .014	*P* = .027
D (control)	4.89 ± 0.33	4.74 ± 0.32	4.45 ± 0.30	4.31 ± 0.28	*P* = .052	–

ANOVA = analysis of variance, SD = standard deviation.

* Within-group comparisons: changes in plaque index from week 1 to week 12 were assessed using a paired *t*-test, with *P*-values adjusted using the Bonferroni correction.

** Between-groups comparisons: differences in plaque index at week 12 among the experimental groups and the control group (group D) were analyzed using one-way ANOVA, followed by a post hoc Tukey test. Adjusted *P*-values were calculated using the Bonferroni correction.

Within-group analyses indicated a significant reduction in PD from week 1 to week 12 in groups B and C (*P* = .026 and *P* = .014, respectively). Group A also showed a decrease in PD over the study period, but the change was comparatively less pronounced (*P* = .034). The control group (group D) experienced a slight decrease in PD; however, this change was not statistically significant (*P* = .052). These findings, reflected in Table 3, indicate that Er:YAG laser treatment, especially at 60 to 80 mJ, offers a clinically meaningful advantage in improving peri-implant tissue health.

### 3.4. Reduction in postoperative pain following Er:YAG laser treatment

Pain levels, assessed using the VAS, are presented in Table 4. While VAS scores were comparable across all groups on day 1, significant reductions were observed by day 3 in groups A, B, and C compared to the control (*P* = .024, *P* = .017, and *P* = .012, respectively). This suggests a rapid and sustained analgesic effect from laser therapy. Within-group analyses revealed a significant reduction in VAS scores from day 1 to day 3 across all experimental groups (*P* = .007 for group A, *P* = .005 for group B, and *P* = .003 for group C). Although a decrease in VAS scores was also observed in the control group, the reduction was comparatively less pronounced (*P* = .017). As detailed in Table 4, these results reinforce the role of Er:YAG laser therapy not only in reducing clinical inflammation but also in improving patient comfort during the early posttreatment period.

**Table 4:** Pain reduction over time in chronic periodontitis patients treated with Er:YAG laser therapy (mean ± SD).

Group	Day 1 (mean ± SD)	Day 2 (mean ± SD)	Day 3 (mean ± SD)	Within-group *P*-value (day 1 vs 3)*	Between-group *P*-value (vs control, day 3)**
A	3.80 ± 0.35	1.45 ± 0.25	0.80 ± 0.20	*P* = .007	*P* = .072
B	3.85 ± 0.33	1.30 ± 0.22	0.75 ± 0.18	*P* = .005	*P* = .051
C	3.90 ± 0.32	1.20 ± 0.21	0.70 ± 0.17	*P* = .003	*P* = .036
D (control)	3.75 ± 0.37	1.85 ± 0.30	1.20 ± 0.28	*P* = .017	–

ANOVA = analysis of variance, SD = standard deviation.

* Within-group comparisons: changes in plaque index from day 1 to day 3 were assessed using a paired *t*-test, with *P*-values adjusted using the Bonferroni correction.

** Between-groups comparisons: differences in plaque index at day 3 among the experimental groups and the control group (group D) were analyzed using one-way ANOVA, followed by a post hoc Tukey test. Adjusted *P*-values were calculated using the Bonferroni correction.

## 4. Discussion

Peri-implantitis is a chronic inflammatory disease caused by microbial infections and remains a significant challenge in implant dentistry.^[2]^ The formation of plaque biofilm is the primary etiological factor, leading to tissue inflammation and progressive bone loss.^[4]^ Conventional mechanical debridement is the most widely used nonsurgical approach for managing peri-implantitis. However, its effectiveness is limited by the implant’s complex surface topography, which can promote bacterial recolonization and recurrent infections.^[10]^ In this study, we evaluated the clinical efficacy of Er:YAG laser therapy for peri-implantitis treatment by testing different energy settings in 40 patients. Our findings suggest that Er:YAG laser therapy improves peri-implant tissue health and reduces inflammation more effectively than conventional drug therapy, particularly at an energy setting of 80 mJ. These results align with previous studies highlighting the benefits of Er:YAG laser therapy, which include its ability to decontaminate implant surfaces efficiently without causing excessive thermal damage to surrounding tissues.^[7–9,11]^

As shown in Table 1, patients treated with Er:YAG laser, particularly in groups B and C, experienced greater reductions in PLI compared to the control group, confirming the laser’s effectiveness in disrupting biofilm accumulation. Our results demonstrated that Er:YAG laser therapy significantly reduced probing depth (PD) and BI compared to baseline levels, with the most pronounced improvements observed in the 80 mJ energy group. The data in Tables 2 and 3 highlight that groups B and C showed statistically significant reductions in BI and PD by week 12 compared to the control, supporting the superior anti-inflammatory effects of higher-energy laser settings over minocycline treatment. This group exhibited superior anti-inflammatory effects over the traditional drug therapy group, reinforcing the notion that laser-assisted therapy can achieve more substantial clinical improvements than conventional nonsurgical approaches. These findings align with previous research, such as the study by Yaneva et al, which reported enhanced clinical attachment levels in chronic periodontitis patients treated with Er:YAG laser compared to those undergoing conventional mechanical debridement.^[12]^ The superior performance of the Er:YAG laser can be attributed to its selective absorption by water molecules, leading to micro-explosions that effectively remove biofilm and necrotic tissue while minimizing collateral thermal damage.

Pain outcomes also reflected a clear benefit of laser therapy. As summarized in Table 4. Pain reduction following laser therapy can be explained by the laser’s ability to modulate inflammatory mediators such as cyclo-oxygenase-2 inhibition and β-endorphin secretion, both of which contribute to reduced postoperative discomfort. These findings are consistent with previous research highlighting that laser therapy reduces pain and inflammation by targeting bacterial biofilm and improving tissue healing.^[13–15]^ Furthermore, Er:YAG laser irradiation has been associated with a photobiomodulatory effect that enhances fibroblast activity and promotes epithelial migration, which may contribute to the observed clinical improvements.^[9,11,16]^

Our study provides new insights into the optimal Er:YAG laser energy settings for peri-implantitis treatment. While previous studies have confirmed the bactericidal effects of Er:YAG lasers,^[7–9,11,12]^ few have specifically evaluated different energy levels and their impact on peri-implant soft tissue health. Our findings indicate that 80 mJ is the most effective setting, balancing bacterial elimination, biostimulatory effects, and patient comfort. This parameter could serve as a guideline for standardizing Er:YAG laser applications in clinical practice.

Moreover, our study emphasizes the sustained effectiveness of Er:YAG laser therapy over time. While PD and BI values remained significantly lower than baseline even after 3 months, the mild rebound observed in Tables 2 and 3 at week 12 suggests the possibility of bacterial recolonization and underscores the limitations of a single-treatment protocol. We recognize that a longer follow-up period would offer more definitive insights into the durability of therapeutic effects and the long-term risk of microbial reestablishment. Therefore, future studies should extend the observation window to 6 months or longer, with periodic assessments to evaluate sustained clinical improvements and the timeline of bacterial recolonization. In addition, adjunctive maintenance strategies (such as combining Er:YAG therapy with systemic or local antimicrobials, or implementing periodic laser reapplications) should be explored to prolong treatment efficacy and reduce the risk of disease recurrence. These considerations may enhance the long-term clinical utility of Er:YAG laser therapy in peri-implantitis management.

Despite its promising results, this study has certain limitations that should be acknowledged. First, we did not include a conventional mechanical debridement arm or an alternative laser system (e.g., Nd:YAG) as comparators. Minocycline gel was chosen as the control because it is a widely used, minimally invasive pharmacologic adjunct and because preliminary data indicated that repeated mechanical instrumentation could cause additional soft-tissue trauma in this patient cohort, whereas Nd:YAG irradiation carries a higher risk of thermal damage to titanium surfaces. Future multi-arm randomized trials directly contrasting Er:YAG therapy with ultrasonic/hand-instrument debridement and with other laser wavelengths are therefore necessary to establish comprehensive clinical guidelines. Although the sample size of 40 patients provided sufficient statistical power to detect treatment effects, the relatively small cohort may limit the generalizability of our findings. Future studies with larger, multicenter patient populations are warranted to validate these results and assess broader applicability. Additionally, while Er:YAG laser therapy effectively reduced microbial load and improved peri-implant tissue health, the observed slight rebound in PD and BI values at 12 weeks suggests that bacterial recolonization remains a challenge. Future studies should explore adjunctive antimicrobial treatments or maintenance protocols, such as combining laser therapy with systemic or local antibiotics, to enhance long-term stability.

Second, variability in implant surface characteristics represents another notable limitation. Differences in implant surface roughness and material composition may influence treatment outcomes. Investigating the efficacy of Er:YAG laser therapy on different implant surface types could provide more tailored treatment recommendations. Additionally, this study focused exclusively on Er:YAG lasers. Future research should compare the efficacy of Er:YAG lasers with other laser types, such as Nd:YAG and diode lasers, to determine which wavelength is most effective for specific peri-implant conditions.

The results of this study suggest that Er:YAG laser therapy could be integrated as a first-line treatment option for peri-implantitis, particularly for patients who are contraindicated for mechanical debridement or pharmacological interventions. The advantages of Er:YAG laser therapy, such as its ability to selectively ablate biofilm without damaging implant surfaces, make it a compelling option for managing peri-implant infections. Moreover, Er:YAG laser therapy can significantly reduce postoperative pain, improve patient comfort, and minimize treatment-related complications, thereby enhancing compliance with periodontal maintenance programs.

To maximize the long-term efficacy of Er:YAG laser therapy, future studies should explore the potential benefits of combining laser treatment with adjunctive antimicrobial agents to mitigate bacterial recolonization. Additionally, periodic laser applications as part of maintenance therapy could help prevent biofilm formation and sustain peri-implant tissue health over extended periods. Further randomized controlled trials with larger patient cohorts should be conducted to confirm these findings and establish standardized protocols for Er:YAG laser use in implant dentistry. Future research should also investigate the long-term effects of Er:YAG laser therapy on peri-implant bone regeneration and soft tissue healing, as well as its potential role in preventing peri-implant disease recurrence.

If optimized, this approach has the potential to redefine peri-implantitis management by reducing the need for more invasive surgical interventions and improving patient outcomes. While current peri-implantitis treatment guidelines remain cautious about the use of lasers, accumulating evidence, including the findings of this study, suggests that Er:YAG laser therapy can be an effective and minimally invasive adjunct to conventional mechanical debridement. As research continues to evolve, Er:YAG lasers are likely to play an increasingly important role in the future of implant dentistry, providing clinicians with an innovative and effective tool for managing peri-implant infections.

## Acknowledgments

The authors would like to thank all the participants in the study, and the research team and colleagues for their valuable contributions, insights, and support during this project.

## Author contributions

**Conceptualization:** Xiaoying Dai, Qingquan Guo.

**Formal analysis:** Xiaoying Dai.

**Investigation:** Xiaoying Dai, Chunmei Liu, Yuxuan Li, Ji Liu, Yimeng Fan.

**Supervision:** Qingquan Guo.

**Writing – original draft:** Xiaoying Dai.

**Writing – review & editing:** Qingquan Guo.
